# A correlation study between brace compliance, in-brace correction and quality of life (QoL) of patients with Adolescent Idiopathic Scoliosis (AIS)

**DOI:** 10.1186/1748-7161-10-S1-O77

**Published:** 2015-01-19

**Authors:** Siu Ling Chan, KMC Cheung, KDK Luk, KWH Wong, Man Sang Wong

**Affiliations:** 1 The University of Hong Kong, Pok Fu Lam, Hong Kong; 2 Queen Mary Hospital, Pok Fu Lam, Hong Kong; 3 The Hong Kong Polytechnic University, Hung Hom, Hong Kong

## Objectives

The objectives of this study were to measure patients' brace compliance; to assess in-brace correction at initial treatment period; to assess quality of life (QoL) of patients with AIS; and to estimate the correlation among patients' brace compliance, in-brace correction and QoL of patients with AIS.

## Material and method

In this prospective correlation study, 55 patients diagnosed with AIS were recruited. All were female and aged 10 years or above when a brace was prescribed, none had undergone prior treatment, and all had a Risser sign of 0-2 and a Cobb angle of 25-40 degree. The patients were examined in 3 consecutive visits with 4-6 months between each visit. The Chinese translated Trunk Appearance Perception Scale (TAPS) (Range 1-5), the Chinese translated Brace Questionnaires (BrQ) (Range 20-100) and the Chinese translated SRS-22 Questionnaires (Range 22-110) were used. In-brace Cobb angle, vertebral rotation and trunk listing were measured. Patients' compliance, in-brace correction and patients' QoL were assessed. To identify the relationship among these three areas, logistic regression model and generalized linear model were used.

## Results

For the compliance measure, a significant difference (p = 0.008) was detected on the TAPS mean score difference between Visits 1 and 2 in the least compliant group (0-8 hours) and the most compliant group (17-23 hours). It was predicted that the TAPS mean score difference of group 1 (0-8 hours) was 0.70 less than that of group 3 (17-23 hours). In addition, a significant difference (p = 0.000) was detected on the BrQ mean score difference between Visits 2 and 3 in the least compliant group (0-8 hours) and the most compliant group (17-23 hours). It was estimated that the BrQ mean score difference of group 1 (0-8 hours) was 16.28 less than that of group 3 (17-23 hours). For the orthosis effectiveness measure, no significant difference was detected between the 3 groups of bracing hours (0-8 hours, 9-16 hours, 17-23 hours) on in-brace correction (below 40% and 40% or above). For the QoL measure, no significant difference was detected between the two different in-brace correction groups (below 40% and 40% or above) on QoL as refected by the TAPS, BrQ and SRS-22r mean scores.

## Conclusion

This study showed a positive and significant relationship between patients' brace compliance and patients' QoL. Neither significant relationship was found between patients' brace compliance and in-brace correction nor between in-brace correction and patients' QoL.

